# How keeping active pays off in the olfactory system

**DOI:** 10.7554/eLife.00326

**Published:** 2012-12-13

**Authors:** Kevin Monahan, Stavros Lomvardas

**Affiliations:** **Kevin Monahan** is in the Department of Anatomy, University of California, San Francisco, USA; **Stavros Lomvardas** is in the Department of Anatomy, University of California, San Francisco, USAstavros.lomvardas@ucsf.edu

**Keywords:** histone, olfactory, epigenetics, Mouse

## Abstract

A protein that is found in the main olfactory epithelium of mice ensures that odour-sensing neurons that are active to have longer lifespans than those that are inactive.

**Related research article** Santoro S, Dulac C. 2012. The activity-dependent histone variant H2BE modulates the life span of olfactory neurons. *eLife* **1**:e00070. doi: 10.7554/eLife.00070

**Image** The protein H2BE in the main olfactory epithelium of a five-week old mouse

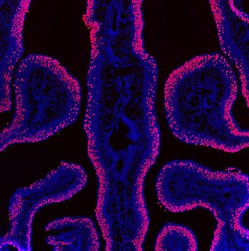

Sensory systems are responsible for gathering information about the world, and relaying it to the brain. Optimal performance is achieved by adapting these systems to suit their environment, and sensory organs employ a variety of astounding anatomical and molecular devices to achieve this (Gracheva et al., 2010, 2011). Most sensory systems, such as the eyes and ears, only need to detect a limited range of stimuli, but the nose must detect and discriminate between the many thousands of different volatile compounds that are produced by a constantly changing environment. Similar to the immune system, the olfactory system needs to be ‘prepared’ to recognize novel odours, while remaining tuned to its current environment.

Odour detection is mediated by G-protein coupled receptors that are expressed by olfactory sensory neurons (Buck and Axel, 1991), with each neuron transcribing one of the thousands of olfactory receptors encoded by the mammalian genome (Chess et al., 1994). Therefore, each neuron responds to a specific set of chemicals. Moreover, olfactory receptor genes are expressed at different frequencies in the mouse olfactory epithelium (that is, some genes are expressed more often than others; Khan et al., 2011), and it is likely that the number of neurons expressing each receptor determines the sensitivity to the corresponding odorants. Writing in *eLife*, Stephen Santoro and Catherine Dulac of Harvard University report that they have identified the protein that orchestrates the frequencies at which different olfactory receptors are expressed, and thus enables the adaptability and plasticity of the olfactory system. This protein, called H2be, is a variant of histone 2b (H2b), which is one of the five main histone proteins that allow the DNA to be efficiently packaged into chromatin in the nuclei of cells.

In an experimental tour de force Santoro and Dulac show that the expression of H2be is highly variable among neurons in the main olfactory epithelium. However, they also show that neurons that express the same olfactory receptor have similar levels of H2be expression, and they go on to demonstrate that there is a correlation between how active the receptor is and the level of H2be expression. In experiments with mice in which one side of the main olfactory epithelium was deprived of olfactory stimulation, high levels of H2be expression were observed on the occluded side. And when the main olfactory epithelium was exposed to odorants, low levels of H2be expression were observed in neurons with receptors that were sensitive to these odorants. It is clear, therefore, that H2be expression is negatively regulated by neuron activity.

Santoro and Dulac then used genetic techniques to show that deletion of the H2be gene led to a change in the number of neurons expressing specific olfactory receptors (with the numbers of some neurons increasing and others decreasing). Reciprocal changes were seen when H2be was overexpressed, but in each case, some receptors became more common on the expense of others, which became less frequent. How does this happen? Olfactory sensory neurons have a finite lifespan, and they are replaced continuously during adulthood. The olfactory receptor expressed by each neuron is selected by a biased random choice mechanism, and subsequently maintained for the life of the neuron (Shykind, 2005). Since a neuron can sense odours only after olfactory receptor choice is made, sensory input can only change the number of neurons for each receptor by causing neurons to switch to a different receptor or by altering the lifespan of neurons expressing a given olfactory receptor. Santoro and Dulac show that the level of H2be expression has a strong effect on lifespan, with knockout of the H2be gene greatly extending the average lifespan of neurons, and overexpression shortening it.

These findings suggest that neurons expressing receptors that detect abundant odorants will live longer than neurons that are mostly inactive, with this bias gradually shaping the population of neurons in the olfactory epithelium towards receptors that detect odorants present within the environment (see Figure 1). This ‘use it or lose it’ model explains previously observed changes in olfactory receptor frequency that occur with age (Rodriguez-Gil et al., 2010), or with exposure to odorants (Jones et al., 2008). Importantly, this model provides an elegant balance between plasticity and adaptation; although the potential to detect a wide range of odours, afforded by the exceptional number of genes for receptors, remains intact, the sensory organ becomes ‘tuned’ and sensitized to odorants relevant to its habitat.

**Figure 1.**
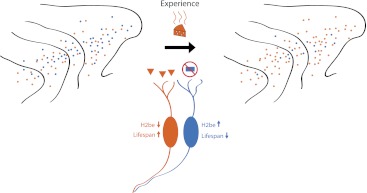
Schematic showing how odour-induced neuronal activity in the nose of a mouse increases the lifespans of frequently activated neurons. Imagine that the mouse can smell orange cheese or blue cheese, so olfactory receptors for orange cheese and blue cheese are scattered across the turbinates in its nose (left). If the mouse is only ever exposed to orange cheese (middle), only those neurons that express the receptor for orange cheese are active, which increases their lifespan and, over time, leads to an increase in the number of receptors for orange cheese (right). However, a small number of receptors for blue cheese remain, and if the mouse was exposed to blue cheese, the number of these receptors would increase. Santoro and Dulac show that a protein called H2be has a central role in this process.

It is clear from this study that other mechanisms also contribute to the activity-dependent changes in the olfactory system because the expression of receptors changes with time in H2be knockout mice (albeit to a lesser extent than in wild-type mice). Santoro and Dulac begin to elucidate the signalling pathway that connects neuronal activity to H2be expression, which may well reveal the identity of other activity-sensitive pathways as well. They show that H2be repression depends on Adcy3, which is an enzyme that generates cyclic AMP (a signalling molecule) in response to odorants. Intriguingly, the olfactory receptors mediate a basal, odorant-independent amount of cyclic AMP signalling, the strength of which varies between olfactory receptors (Imai and Sakano, 2008). This suggests the possibility that those neurons expressing olfactory receptors with high basal cyclic AMP signalling are ‘immune’ to the environmental input, which would explain why removing sensory input amplified, rather than reduced, differences in the frequency of olfactory receptor choice. If this is the case, one could only speculate about the value of olfactory receptors with high basal activity, since these might have emerged through selection mechanisms that assured their ample representation.

It is unclear how H2be regulates neuronal longevity or how a difference of 5 amino acids from canonical H2b affects gene expression. Importantly, H2be is efficiently incorporated into nucleosomes, preferentially within transcribed genes, thereby mediating a rather global change in chromatin composition. One intriguing possibility is that H2be may be subject to different post-translational modifications than canonical H2b. Indeed, Santoro and Dulac show that lysine 5 of H2be is not acetylated or methylated, unlike what happens with canonical H2b, and that the level of the these post-translational modifications in olfactory sensory neurons varies with H2be levels. Since these modifications have been positively correlated with transcription (Wang et al., 2008) it is intriguing to think that global transcription is less efficient in neurons with high H2be levels. Whether this is the case, and how such a global but moderate effect in gene expression could affect neuronal lifespan, will surely be the subject of future experiments. The work of Santoro and Dulac also raises an interesting question about the brain, an organ in which plasticity is achieved by changing synapses rather than eliminating neurons: is abnormal expression of H2be in the brain the cause of neuronal death in various neurodegenerative disorders?
